# Case Report: Intracranial hemophilic pseudotumor mimicking an aggressive neoplasm: a rare skull-invasive presentation

**DOI:** 10.3389/fsurg.2025.1709321

**Published:** 2026-01-12

**Authors:** Moksada Regmi, Junyi Liu, Shikun Liu, Ying Xiong, Zihan Zhao, Xu Zhang, Chenlong Yang

**Affiliations:** 1 State Key Laboratory of Vascular Homeostasis and Remodeling, Department of Neurosurgery, Peking University Third Hospital, Peking University, Beijing, China; 2 Center for Precision Neurosurgery and Oncology of Peking University Health Science Center, Peking University, Beijing, China; 3 Peking University Health Science Center, Beijing, China; 4 Center for Oculocranial Pressure Instability Disorders, Henan Academy of Innovations in Medical Science (AIMS), Zhengzhou, China; 5 Shenzhen Graduate School, Peking University, Shenzhen, China

**Keywords:** hemophilic pseudotumor, intracranial hemorrhage, factor VIII deficiency, neurosurgical resection, skull lesion

## Abstract

Hemophilic pseudotumor is a rare complication of hemophilia in which a chronic, expanding hemorrhagic mass erodes adjacent bone; intracranial presentations are exceedingly uncommon and can mimic neoplasms. We describe a 36-year-old man with severe hemophilia A who developed a five-year, progressively enlarging left frontoparietal swelling with worsening right-sided hemiparesis. MRI demonstrated a heterogeneously enhancing extra-axial lesion destroying the left cranial vault with “mushroom-like” extracranial extension, marked mass effect, and midline shift, initially interpreted as a possible meningioma. Given progressive deficits, the patient underwent resection under intensive factor VIII replacement. Intraoperatively, a 10 × 10 × 5 cm encapsulated lesion containing organizing clot and fibrous tissue was excised *en bloc*; the skull defect was reconstructed in a staged procedure. Histopathology confirmed hemophilic pseudotumor. Postoperatively, hemiparesis improved markedly; a small epidural hematoma was managed conservatively. At 12 months, MRI showed no recurrence and the patient remained neurologically intact on prophylactic factor VIII. This case highlights the need to include hemophilic pseudotumor in the differential diagnosis of skull lesions in patients with hemophilia and underscores the value of early recognition and multidisciplinary management—particularly meticulous perioperative hemostatic support—to enable safe resection and excellent outcomes.

## 1 Background

Hemophilic pseudotumor is an uncommon but serious complication of hemophilia, occurring in approximately 1%–2% of patients with severe factor VIII or IX deficiency (1). It is essentially a chronic expanding hematoma caused by repeated bleeding into a confined space, leading to a mass that can progressively enlarge and cause bone destruction or resorption (1). Most hemophilic pseudotumors develop in the appendicular skeleton (e.g., femur, pelvis) where they cause erosive bone lesions (2). Cranial (intracranial) hemophilic pseudotumors are exceedingly rare, with only a handful of cases reported in the literature (3). Such lesions can clinically and radiologically mimic true tumors, making diagnosis challenging. In the contemporary era, advanced hemophilic pseudotumors are now rarely seen in patients with consistent access to modern prophylaxis, as current World Federation of Hemophilia (WFH) guidelines recommend early and sustained factor replacement for all individuals with severe hemophilia (4). However, significant global disparities in access to prophylaxis mean that patients in many regions remain at risk of developing such complications. We present a case of a giant intracranial hemophilic pseudotumor with skull invasion that was initially mistaken for an aggressive neoplasm. This case underscores the importance of recognizing this rare entity and highlights the management principles required for a favorable outcome.

## 2 Case presentation

A 36-year-old man with known hemophilia A (factor VIII activity <1%, severe) presented with a five-year history of a progressively enlarging swelling over his left forehead and scalp. The mass began as a small, painless “lump” in the left frontoparietal region, which he noticed after minor head bumps. Over the ensuing years it grew slowly but steadily. The patient experienced intermittent headaches and, in the last year, had developing weakness of the right side of his body. He denied any major head trauma or external bleeding. There was no history of seizures or incontinence. Prior evaluations at outside hospitals had included a head CT scan, which showed a large left cranial vault lesion with bone erosion; this was initially suspected to be a meningioma. Because of concern for surgical bleeding risk given his hemophilia, he had been managed conservatively with factor supplementation alone at those institutions. Approximately three months before the current admission, he first presented to our center with marked enlargement of the left frontoparietal swelling. After optimization, he underwent navigation-assisted resection of the lesion and was discharged about one month later with transient improvement. In the weeks following discharge, the left frontotemporoparietal–occipital region again became progressively swollen (to approximately 10 × 10 × 5 cm) without skin breakdown, prompting re-admission for further evaluation and definitive management.

On examination at the current admission, the patient was alert with normal cognitive function. There was a visible bulge over the left frontoparietal skull measuring approximately 10 × 10 cm. The mass was firm on palpation, non-tender, and fixed to the underlying bone. The overlying scalp was intact without redness or ulceration. Neurologic exam revealed a right-sided hemiparesis (Medical Research Council grade 4/5 strength in the right arm and leg) with brisk reflexes on the right. There were no cranial nerve deficits, and sensation was intact. Fundoscopy showed no papilledema.

### 2.1 Studies

**Figure 1:**
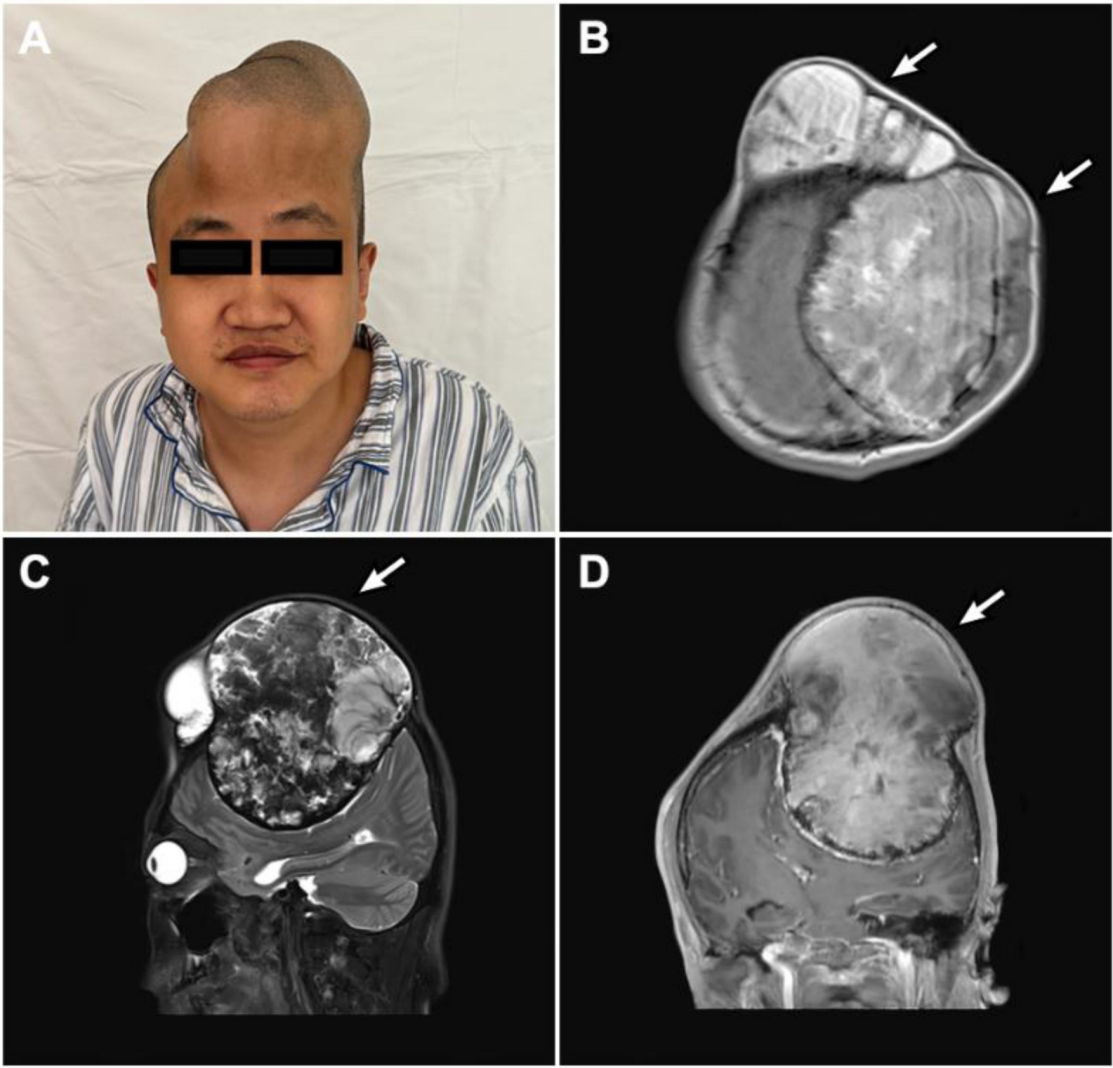
Clinical and radiological features. **(A)** Clinical photograph showing a large, firm cranial bulge over the left frontoparietal region; **(B)** axial T1-weighted MRI demonstrating a massive heterogeneous lesion with skull invasion and midline shift; **(C)** T2-weighted sagittal MRI showing craniocaudal tumor expansion and extracranial extension; **(D)** gadolinium-enhanced coronal MRI depicting the “mushroom-like” morphology and bony remodeling.

Magnetic resonance imaging (MRI) of the brain demonstrated a massive extra-axial lesion centered in the left frontoparietal region, with extensive destruction of the adjacent skull. On T1-weighted sequences the lesion was heterogeneous in signal with areas of iso- to hyperintensity, and it showed heterogeneous enhancement after gadolinium. T2-weighted and FLAIR images revealed a complex mix of hyperintense and hypointense components with a fluid–fluid level appearance, consistent with chronic blood products at different stages. The lesion extended both intracranially and extracranially: it had formed a “mushroom-like” extracranial expansion through a defect in the cranium, producing a prominent subgaleal mass outside the skull (Figure 1). Significant vasogenic edema in the adjacent left frontal lobe was present. There was a marked mass effect with compression of the left frontal lobe and a midline shift of approximately 1.5 cm to the right. The ventricles on the left were effaced. These imaging features (an extra-axial skull-based mass with bone erosion and mixed signal intensities) raised suspicion of a chronic expanding hematoma (pseudotumor) rather than a typical neoplasm. In particular, the absence of a clear dural attachment and the presence of fluid levels and calcified rims favored a hemorrhagic lesion. Given the patient's hemophilia history, an intracranial hemophilic pseudotumor became the leading diagnosis, although invasive skull tumors (such as an atypical meningioma or hemangiopericytoma) remained in the differential.

Laboratory tests on admission were notable for a factor VIII activity <1% (baseline), confirming severe hemophilia A. He had a prolonged activated partial thromboplastin time (aPTT), as expected, which corrected with mixing studies. There was no evidence of factor VIII inhibitor (inhibitor screen was negative). Other labs, including complete blood count and metabolic panel, were within normal limits aside from mild anemia (hemoglobin 11.8 g/dL). Following initiation of factor VIII replacement, aPTT rapidly normalized and remained within the institutional reference range; around the end of the first postoperative week, his factor VIII activity was 172.4%, confirming adequate replacement.

### 2.2 Management

After multidisciplinary discussion, we proceeded with surgical resection of the mass to relieve pressure on the brain and obtain a definitive diagnosis. In accordance with the WFH guidelines for the management of hemophilia and institutional hemophilia-surgery protocols, the patient received intensive recombinant factor VIII (*Kangsiping*, human coagulation factor VIII) replacement. During the preoperative optimization phase (hospital days −5 to −1), he was given 1,200 IU intravenously every 12 h (Q12H). On the final 24 h before surgery (hospital day −1 through the night before day 0), the dose was escalated to 1,600 IU IV Q12H. On the morning of surgery (day 0), an additional 1,600 IU bolus was administered intravenously immediately before incision to achieve a target activity >100%. Postoperatively, 1,600 IU IV Q12H was continued from the evening of day 0 through postoperative day (POD) 7 to maintain FVIII trough levels >80% in the first week and >50% during early wound healing. The detailed dosing schedule is summarized in Table 1.

**Table 1:** Peri-operative and early post-discharge factor VIII replacement regimen.

Phase	Day(s) relative to surgery	FVIII dose per infusion	Frequency & route
Preoperative optimization	−5 to −1	*Kangsiping* 1,200 IU	IV Q12H (2×/day)
Intensified pre-op	−1–0	*Kangsiping* 1,600 IU	IV Q12H (2×/day)
Pre-incision bolus	Day 0	*Kangsiping* 1,600 IU	Single IV bolus
Early postoperative maintenance	0–7	*Kangsiping* 1,600 IU	IV Q12H (2×/day)
Late postoperative/early discharge period	>7	*Kangsiping* 1,600 IU	IV Q12H

An additional 1,200 IU dose was ordered on the evening of day 0 but was documented as discontinued before administration and was not given.

Low-molecular-weight heparin thromboprophylaxis was also used cautiously: enoxaparin was administered once in a low dose (∼8.5 mg) on the day of surgery, followed by a single 40 mg subcutaneous dose on POD 2, after confirming stable postoperative imaging and satisfactory hemostasis.

Surgery was performed under general anesthesia with neurosurgical and hematology teams in attendance. The patient was placed supine with the head neutral and slightly rotated, and a transverse fusiform scalp incision of approximately 6 cm was made over the most prominent part of the frontoparietal mass. After standard preparation and draping, the skin and subcutaneous tissues were opened, revealing a cavity filled with old, dark-red clot consistent with chronic hematoma. This cavity communicated with the underlying skull defect and epidural space corresponding to the pseudotumor seen on imaging. Organizing clot and fibrous tissue were carefully evacuated and the pseudotumor capsule was removed as completely as possible. Meticulous hemostasis was achieved, and a subgaleal drain was left in place before layered closure with silk sutures. The patient remained hemodynamically stable throughout the procedure, and the specimen was sent for histopathological examination.

Histopathological examination of the resected material (scalp and skull-associated tissue) confirmed the diagnosis of hemophilic pseudotumor. Macroscopically, the specimen consisted of irregular skin and subcutaneous tissue with underlying bone fragments measuring roughly 5 × 1.6 × 1.5 cm. Microscopically, the epidermis was unremarkable, while the dermis and subcutaneous tissue showed dense chronic inflammatory cell infiltration with extensive areas of old hemorrhage and blood degradation products. No neoplastic cells were identified. These findings, in conjunction with the clinical and radiologic picture of an expansile skull lesion, were consistent with an organizing chronic hematoma/hemophilic pseudotumor rather than a true tumor.

### 2.3 Postoperative course

**Figure 2:**
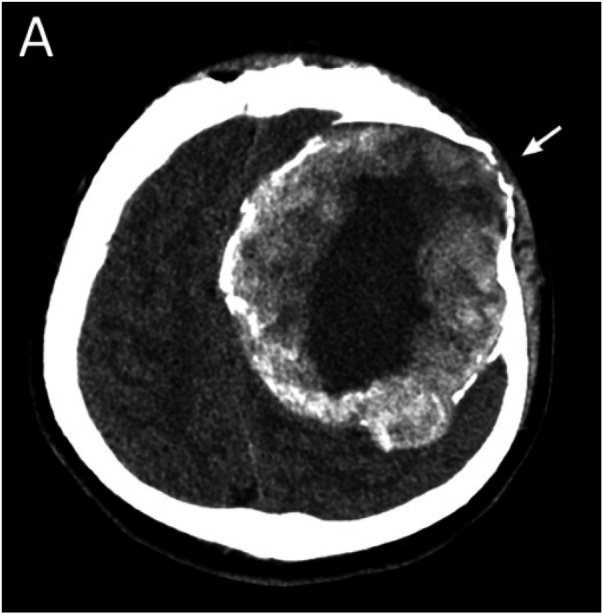
Early postoperative CT after pseudotumor excision. Axial CT shows the left frontoparietal postoperative cavity and skull defect (arrow).

Immediate postoperative imaging (Figure 2) confirmed decompression of the left hemisphere with no residual mass. The patient was monitored in the intensive care unit with continued factor VIII supplementation and close neurological observation. His postoperative aPTT remained within normal range under replacement therapy. He showed immediate improvement in his right-sided strength after surgery—by postoperative day 2, his hemiparesis had nearly resolved (5/5 power in the right leg and 4+/5 in the right arm). On day 3, a routine CT scan of the head showed a small epidural hematoma under the edge of the craniectomy site. Fortunately, the hematoma was limited and the patient remained asymptomatic; this was managed conservatively with continued factor therapy and bed rest. The subgaleal drain was removed on day 4 with minimal output. He completed a two-week course of antibiotics for surgical prophylaxis and continued factor VIII infusions to maintain a trough level >50% during wound healing. The remainder of his hospital stay was uneventful, and he was discharged on postoperative day 14 with significant neurologic recovery. At discharge, he had only a mild residual right hand clumsiness. On follow-up at 12 months after surgery, the patient was doing well. He experienced no neurological deficits and had returned to normal daily activities.

## 3 Discussion

Intracranial hemophilic pseudotumor is an extraordinarily rare entity and can easily be mistaken for a true tumor, as initially happened in this case. In a patient with hemophilia, however, any unusual progressive bony swelling or mass lesion should prompt consideration of a pseudotumor in the differential diagnosis. Misdiagnosis as a neoplasm (for example, meningioma or bone tumor) can lead to delayed or inappropriate management. In our patient, the lesion's imaging characteristics—notably the heterogeneous signal with blood-fluid levels and the extensive bone erosion—were retrospectively recognized as classic for a chronic hemorrhagic pseudotumor rather than a solid tumor. Radiologic findings of pseudotumors often include large soft-tissue masses with adjacent bone destruction and can mimic neoplastic lesions like giant cell tumors, aneurysmal bone cysts, and soft tissue sarcomas (5). This case highlights that greater awareness is needed among clinicians to identify such features, especially in known hemophilia patients.

Hemophilic pseudotumors develop through repeated bleeding episodes that are not adequately resorbed, resulting in an encapsulated mass of clotted blood that expands over time. The expanding hematoma gradually erodes local bone and soft tissue, hence the term “pseudotumor” for its tumor-like mass effect. Minor trauma or even spontaneous hemorrhage can initiate the formation of these lesions. In fact, pseudotumors may initially be asymptomatic or stable for years before presenting with pain due to mass effect, nerve compression, or pathological fractures. In our case, the patient's recollection of a minor head injury preceding the onset of the scalp lump is consistent with this pathogenesis. Over years, the lesion attained an enormous size due to ongoing bleeding into the cavity, facilitated by long-standing on-demand rather than continuous prophylactic factor treatment prior to referral.

Importantly, advanced pseudotumors of this size are now considered largely preventable in settings where guideline-concordant prophylaxis is routinely available. The WFH guidelines emphasize that patients with severe hemophilia should be placed on regular prophylactic factor replacement from early childhood to prevent joint disease and other chronic bleeding complications. Nevertheless, access to factor concentrates and structured prophylaxis remains uneven worldwide. Our patient had not been on continuous prophylaxis before his initial presentation and had limited access to factor VIII in the past, a context that likely allowed the pseudotumor to develop and later to recur after the first operation.

Given the potential for significant morbidity, the management of hemophilic pseudotumor requires a careful, case-by-case approach. Small or asymptomatic pseudotumors (especially in locations like long bones) may sometimes be managed conservatively with clotting factor replacement and close observation. In some instances, adjunctive radiotherapy has been used to help sterilize and shrink the pseudotumor when surgery is not feasible. In cases where surgical excision is not an option, combining radiotherapy with factor replacement has shown some success in managing distal pseudotumors, particularly in extremities. In fact, for hemophilic bone pseudotumors located in the skull (or in anatomically delicate areas), a combination of factor replacement therapy and radiotherapy has been suggested as a conservative alternative if surgical excision would be high-risk (6). However, surgical removal remains the preferred treatment in the majority of cases, particularly for large pseudotumors causing significant pressure effects or functional impairment (6). Surgical excision allows immediate decompression of affected structures and provides tissue for definitive diagnosis. This can be critical in distinguishing pseudotumors from true neoplasms, as failure to do so may lead to inappropriate oncologic management (5). In our patient, given the severe mass effect and hemiparesis, there was a clear indication for surgery. The successful outcome in this case supports the literature that with proper technique and hemostatic control, surgery can be curative for cranial hemophilic pseudotumors (3).

Crucial to the surgical management is a multidisciplinary strategy that addresses the bleeding risk. Close coordination with a hematologist is required to ensure appropriate factor levels are maintained before, during, and after the operation. Current guidelines and experience indicate that factor levels should be raised to 80%–100% of normal prior to major surgeries in hemophiliacs and sustained for at least 7–14 days postoperatively (7). One study found that perioperative management following these protocols significantly reduced the risk of surgical bleeding complications and improved outcomes in patients with hemophilic pseudotumors (8). In this case, adherence to such a regimen enabled the surgical team to operate with acceptable hemostasis. We did encounter a small epidural bleed postoperatively, which underscores that even with adequate factor replacement, careful monitoring is necessary. Fortunately, no major hemorrhagic complication occurred, and the small hematoma was managed non-operatively. The use of perioperative factor concentrate and, if needed, fibrin glue or other hemostatic adjuncts during surgery can mitigate intraoperative bleeding risks. Additionally, performing the surgery with modern neurosurgical techniques (e.g., microscope, navigation) allowed precise resection while avoiding injury to normal brain tissue. Our detailed peri-operative factor VIII regimen (Table 1) show how guideline-based dosing can be implemented in practice and may serve as a practical template for similar cases.

Histologically, hemophilic pseudotumors are benign lesions composed of organized hematoma with surrounding fibrous reaction and often calcifications. In our case, the histology showed organized hematoma with fibrosis, which is characteristic of pseudotumors, distinguishing them from malignant tumors such as chondrosarcoma and synovial sarcoma, which can mimic these lesions clinically and radiologically. As in our patient's case, there is no evidence of neoplastic cells—distinguishing it from cancerous tumors. Confirming the pathology is important because it justifies the continuation of a non-oncologic management pathway (focused on controlling bleeding) and avoids unnecessary oncologic therapies. In previously reported cranial pseudotumors, the histopathology has uniformly shown chronic organized clots, sometimes described as “chocolate cysts” due to the brown, liquefied blood contents. This finding was echoed in our case and is diagnostic when correlated with the clinical context of hemophilia.

Outcomes for patients with hemophilic pseudotumor have improved significantly with advances in care. Surgical series and case reports indicate that, when complete resection is achieved and adequate factor coverage is provided, patients often have good recovery of function and a low risk of recurrence. For example, one literature report noted no recurrence of a cranial pseudotumor at 3-year follow-up after total excision combined with rigorous factor replacement therapy (3). Our patient similarly remained recurrence-free one year after surgery. Long-term prophylactic factor administration is a key part of preventing pseudotumor recurrence or new pseudotumor formation elsewhere (6). The patient in this case was placed on a high-dose prophylaxis regimen postoperatively, which likely contributed to his durable remission. In contrast, inadequate factor control could allow continued bleeding and regrowth of a pseudotumor. There have been instances of lesions recurring, particularly if only partially resected or if the patient develops a factor inhibitor that complicates replacement therapy. Thus, diligent follow-up is required. In our patient, factor VIII inhibitor testing was negative at baseline, and there has been no clinical evidence of inhibitor development during 12 months of intensive replacement and secondary prophylaxis.

This case adds to the very limited pool of reported intracranial hemophilic pseudotumors. It demonstrates that even a very large, skull-invasive pseudotumor can be managed successfully with modern therapy. Key lessons include maintaining a high index of suspicion for pseudotumor in hemophiliacs with unusual masses, the importance of collaborative care (neurosurgeons working closely with hematologists), and the effectiveness of surgical intervention when indicated. For neurosurgeons, recognizing the imaging hallmarks of a hemophilic pseudotumor (heterogeneous hemorrhagic lesion with bone erosion) can prompt timely hematologic evaluation and preoperative optimization. For hematologists, this case underlines the necessity of aggressive factor replacement and possibly immunologic therapy (to prevent inhibitors) when managing such a patient.

## 6 Conclusions

Intracranial hemophilic pseudotumor is a rare but important diagnostic consideration in hemophilia patients presenting with skull lesions. Early diagnosis can prevent mismanagement, and timely surgical excision—in conjunction with appropriate factor VIII/IX replacement—can lead to excellent outcomes. In line with modern WFH guidelines, long-term prophylactic factor replacement is essential both to prevent the initial development of pseudotumor and to avoid recurrence after treatment. This case exemplifies that a multidisciplinary approach can achieve complete tumor removal and neurological recovery without significant hemorrhagic complications. Ongoing prophylactic factor therapy remains essential after treatment to prevent recurrence. Increased awareness of this condition will help clinicians promptly recognize and treat future cases, thereby avoiding permanent disability from untreated mass effect.

## Data Availability

The original contributions presented in the study are included in the article/Supplementary Material, further inquiries can be directed to the corresponding author.
